# scGSCA: gene-set differential co-expression analysis for single-cell RNA sequencing data

**DOI:** 10.64898/2026.10.05.756579

**Published:** 2026-10-12

**Authors:** Zhuoli Jin, Rebecca Irlmeier, Jie Ping, Yuan Wang, Yan Guo, Fei Ye

## Abstract

Understanding disease-associated pathway coordination requires distinguishing phenotype-related co-expression changes from differences across cellular contexts while accounting for cells sampled from the same subject. Bulk gene-set co-expression methods do not directly address the sparse counts, single-cell distributional noise, subject-level dependence, and cell-type heterogeneity of single-cell RNA sequencing (scRNA-seq) data. We developed single-cell gene-set co-expression analysis (scGSCA), a framework for pathway-level differential co-expression analysis in scRNA-seq data. scGSCA fits gene-wise zero-inflated negative binomial mixed models with subject-level random effects and computes gene-set co-expression statistics from quantile residual correlations. It provides an overall differential gene-set co-expression analysis and an interaction test to assess whether the co-expression pattern between two cell types varies by sample phenotype. We applied scGSCA to tumor–normal gastric, breast-subtype, and lung–skin comparisons with matched bulk and scRNA-seq data. The overall test identified signals not captured in bulk analysis, including Axon guidance in gastric cancer and estrogen-response, IL6-JAK-STAT3, and TNF- *α* /NF-κB pathways in breast cancer. The interaction test further identified co-expression contrasts across cell types. scGSCA extends gene-set co-expression analysis to single-cell data while retaining subject-level structure and cell-type-specific context. scGSCA is freely available as an open-source R package.

